# Using health facility-based serological surveillance to predict receptive areas at risk of malaria outbreaks in elimination areas

**DOI:** 10.1186/s12916-019-1482-7

**Published:** 2020-01-28

**Authors:** Henry Surendra, Supargiyono, Riris A. Ahmad, Rizqiani A. Kusumasari, Theodola B. Rahayujati, Siska Y. Damayanti, Kevin K. A. Tetteh, Chetan Chitnis, Gillian Stresman, Jackie Cook, Chris Drakeley

**Affiliations:** 1 Department of Infection Biology, London School of Hygiene and Tropical Medicine, London, WC1E 7HT UK; 2 Centre for Tropical Medicine, Faculty of Medicine, Public Health and Nursing, Universitas Gadjah Mada, Jl. Medika, Yogyakarta, 55281 Indonesia; 3 Department of Parasitology, Faculty of Medicine, Public Health and Nursing, Universitas Gadjah Mada, Sekip Utara, Yogyakarta, 55281 Indonesia; 4 Department of Biostatistics, Epidemiology and Population Health, Faculty of Medicine, Public Health and Nursing, Universitas Gadjah Mada, Sekip Utara, Yogyakarta, 55281 Indonesia; 5 District Health Office of Kulon Progo, Jln. Suparman No 1, Wates, 55611 Indonesia; 6 Institut Pasteur, Paris, France; 7 MRC Tropical Epidemiology Group, Department of Infectious Disease Epidemiology, London School of Hygiene and Tropical Medicine, London, WC1E 7HT UK

**Keywords:** Serology, Surveillance, Mapping, Malaria, Elimination

## Abstract

**Background:**

In order to improve malaria burden estimates in low transmission settings, more sensitive tools and efficient sampling strategies are required. This study evaluated the use of serological measures from repeated health facility-based cross-sectional surveys to investigate *Plasmodium falciparum* and *Plasmodium vivax* transmission dynamics in an area nearing elimination in Indonesia.

**Methods:**

Quarterly surveys were conducted in eight public health facilities in Kulon Progo District, Indonesia, from May 2017 to April 2018. Demographic data were collected from all clinic patients and their companions, with household coordinates collected using participatory mapping methods. In addition to standard microscopy tests, bead-based serological assays were performed on finger-prick bloodspot samples from 9453 people. Seroconversion rates (SCR, i.e. the proportion of people in the population who are expected to seroconvert per year) were estimated by fitting a simple reversible catalytic model to seroprevalence data. Mixed effects logistic regression was used to examine factors associated with malaria exposure, and spatial analysis was performed to identify areas with clustering of high antibody responses.

**Results:**

Parasite prevalence by microscopy was extremely low (0.06% (95% confidence interval 0.03–0.14, *n* = 6) and 0 for *P. vivax* and *P. falciparum*, respectively). However, spatial analysis of *P. vivax* antibody responses identified high-risk areas that were subsequently the site of a *P. vivax* outbreak in August 2017 (62 cases detected through passive and reactive detection systems). These areas overlapped with *P. falciparum* high-risk areas and were detected in each survey. General low transmission was confirmed by the SCR estimated from a pool of the four surveys in people aged 15 years old and under (0.020 (95% confidence interval 0.017–0.024) and 0.005 (95% confidence interval 0.003–0.008) for *P. vivax* and *P. falciparum*, respectively). The SCR estimates in those over 15 years old were 0.066 (95% confidence interval 0.041–0.105) and 0.032 (95% confidence interval 0.015–0.069) for *P. vivax* and *P. falciparum*, respectively.

**Conclusions:**

These findings demonstrate the potential use of health facility-based serological surveillance to better identify and target areas still receptive to malaria in an elimination setting. Further implementation research is needed to enable integration of these methods with existing surveillance systems.

**Electronic supplementary material:**

The online version of this article (10.1186/s12916-019-1482-7) contains supplementary material, which is available to authorized users.

## Background

Transforming malaria surveillance into a core intervention is one of the three pillars of the WHO global technical strategy for malaria elimination [1]. As transmission declines, malaria risk becomes more heterogeneous and is often clustered in specific localities or populations [2, 3]. Identifying areas of ongoing infection or areas at risk of outbreaks is important to ensure that control strategies can be deployed in the most efficient manner [4–6]. In many Southeast Asian settings, surveillance becomes more challenging with the presence of multi-species infections combined with the difficulty of identifying where, and in which populations, residual transmission might be occurring [7, 8].

In many countries, surveillance has focused on passive case detection performed via health facilities [9, 10]. However, innovative additional strategies are needed in countries nearing elimination as malaria cases become increasingly rare and disproportionately affect high-risk populations, who may not utilise public health facilities [10]. Studies suggest that passive surveillance will miss a large proportion of asymptomatic and sub-microscopic infections present in the community [8, 11, 12] and may also not optimally capture imported infections occurring in temporary visitors who may be unable or unlikely to visit a health facility. Effectively targeting both of these groups is likely to hasten progress toward elimination.

Resurgence of malaria is often associated with imported infections and/or *P. vivax* relapsing infections in areas that remain highly receptive to malaria [13–16]. Studies have demonstrated the usefulness of spatially referenced entomological data to characterise the heterogeneity of malaria receptivity in areas approaching elimination to prevent outbreaks in the future [17–19]. However, entomological surveillance can often be logistically challenging in low transmission areas due to the difficulty of catching meaningful numbers of mosquitoes. An alternative approach is to identify areas where the population show evidence of current or previously high malaria exposure. This can be done using serological markers of infection and identifying populations with higher than average anti-malaria antibodies [20–23]. Serological measures are a sensitive tool to estimate current and previous transmission intensity in a population and their use has been particularly well validated in low transmission areas where the sensitivity of parasitological tools is inadequate [24–27]. However, these studies used community-based cross-sectional surveys that often require large resources to visit households for collecting samples and household global positioning system coordinates to map the transmission risk. In order to further reduce logistical constraints, convenience sampling approaches targeting health facility attendees can be used to estimate and map risks in a population when household surveys are not feasible [28] and has been shown to be a good proxy for malaria transmission in the community [29]. Moreover, the simple addition of a geolocation approach to remotely record the residence of health facility attendees in the survey [30] allows for rapid assessment of the micro-epidemiology of malaria cases in the community and could help to identify geographical foci of exposure.

Indonesia is one of countries facing challenges in eliminating both *Plasmodium falciparum* and *Plasmodium vivax* infections. Previous studies in Indonesia suggest that the current diagnostic sensitivity (microscopy and rapid diagnostic test (RDT)) and timeliness of transmission measurement are not sufficient to describe and predict decreasing numbers of cases and potential outbreaks in low transmission areas striving for elimination [31–33]. The risk of outbreaks is high where there are larger numbers of migrants or travellers [31, 34–36] and/or where residents with asymptomatic infections are not actively seeking treatment for malaria [37–45]. Therefore, surveillance systems need to be improved to better locate and target infections and further reduce transmission [32, 46]. This study evaluated the use of serology, geolocation tools, and repeated health facility-based surveys for capturing malaria transmission dynamics in conjunction with existing surveillance system in an area conducting elimination in Indonesia.

## Methods

### Study setting

**Fig. 1:**
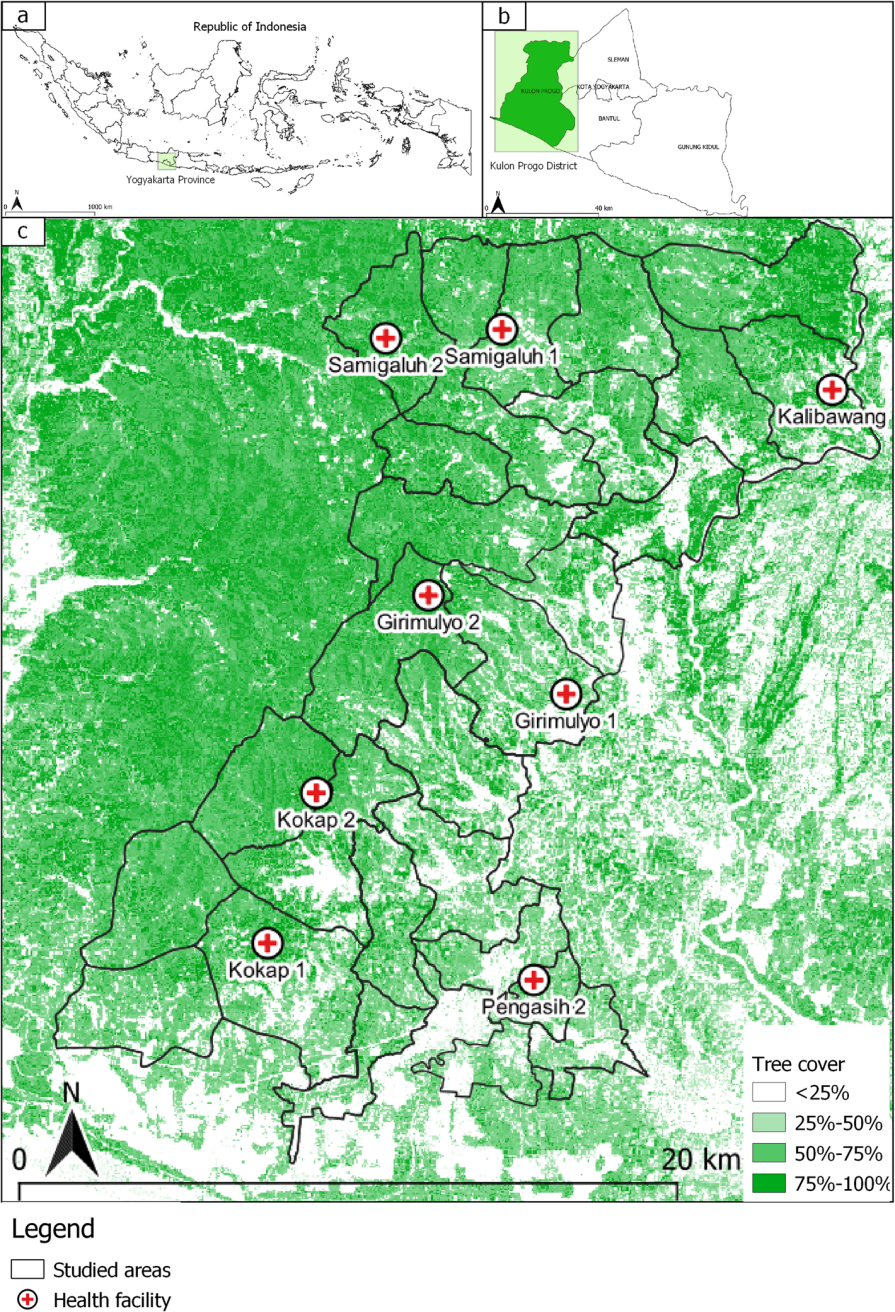
Maps showing the location of Yogyakarta Province in Indonesia (**a**), location of Kulon Progo District in Yogyakarta Province (**b**), and the location of eight studied health facilities in Kulon Progo District (**c**). Tree cover data, derived from classified Landsat imagery at 30-m resolution, were obtained from Hansen et al. [57]

Indonesia has the second highest burden of malaria in the Southeast Asia region, with an estimated 16 million people (~ 6% of the population) living in high-risk areas [47]. All species of Plasmodium have been reported in Indonesia with the majority of infections caused by *P. falciparum* and *P. vivax* [35, 48–51]. Malaria transmission is highly heterogenous [52, 53], with large areas being transmission free, leading to a governmental target of achieving malaria elimination across the country by 2030 [46]. This study was conducted in Kulon Progo District, Yogyakarta Province, Indonesia, located on the south coast of Java Island. Kulon Progo is one of the few remaining foci of malaria transmission on Java Island, Indonesia (Fig. 1). The study site consists of 12 sub-districts (586 km^2^ in total) with a population of approximately 430,500 people in 2016. Each district has at least one public health facility (21 in total). Malaria transmission is concentrated in the forested hillside area that border with other endemic areas of Central Java Province [54]. Transmission occurs during the wet season between August and December, with very low or zero cases during the other months. Based on routine passive data recorded in local health facilities, there was a significant decline in malaria annual parasite incidence from 0.48 per 1000 population in 2012 to 0.22 per 1000 population in 2016. Eight health facilities in 5 sub-districts where *P. falciparum* and/or *P. vivax* transmission was ongoing were chosen as study sites. *Anopheles maculatus* and *Anopheles balabacencis* are the main malaria vectors in Kulon Progo [55].

### Survey design and data collection

The study population included all attendees of the eight selected public health facilities. Surveys were conducted quarterly during the period of May 2017 to April 2018. Each survey continued until the minimum sample size was met. The sample size calculation was performed using methods specific for estimating antibody seroconversion rates (SCR, i.e. the proportion of people in the population who are expected to seroconvert per year) [56]. The SCR to either *P. falciparum* apical membrane antigen 1 (PfAMA1) or merozoite surface protein 1 (PfMSP-1-_19_) in Kulon Progo was expected to be lower than the SCR reported in the neighbouring pre-elimination setting, Purworejo District, Indonesia (SCR 0.019 (95% CI 0.015–0.022)). Therefore, a minimum sample size of 248 individuals per facility was set to ensure an antibody SCR of 0.0036 could be estimated with a precision level of +/− 0.0018.

Finger-prick blood samples were collected as dried bloodspots together with thick and thin blood smears from all consenting participants attending the facilities. Patients who were very ill and required urgent care, and children < 6 months of age were excluded. Data on age, gender, axillary temperature, patient (versus accompanying person) status, permanent residence, travel behaviour, occupation, bed net use and current symptoms or reasons for attending the clinic were collected. Fever status was defined as having axillary temperature > 37.5 °C and/or reported having fever in the previous 24 h of sample collection. Participants were asked to geolocate their household using high-resolution digital offline maps via the open source GeoODK. The validation of this mapping approach was performed at the beginning of our first survey and has been reported in Fornace et al. [30]. All data were collected via interview using open data kit (https://opendatakit.org/) on tablets (Samsung Galaxy Tab 3 SM-T210). Demographic data on reported cases, surveillance (passive and reactive case findings) and control programme activities were collected from the District Health Office of Kulon Progo in between every serological survey. Data on the age distribution of the population in the study area was obtained from the 2016 census published by the Central Agency on Statistics of Kulon Progo. Tree cover data, derived from classified Landsat imagery at 30 m resolution, were obtained from Hansen et al. [57].

### Laboratory methods

Thick and thin blood smears were read by trained health facility lab technicians at each facility. Bloodspot samples were tested against a panel of *P. falciparum* and *P. vivax* antigens including apical membrane antigen 1 (PfAMA1; PvAMA-1), merozoite surface protein 1 (PfMSP-1-_19_; PvMSP-1-_19_), erythrocyte binding protein (PvEBP), reticulocyte binding protein 1a [amino acids 160–1170] (PvRBP1a) and reticulocyte binding protein 2b [amino acids 161–1454] (PvRBP2b) using a bead-based assay as described by Wu et al. [58] and read using Luminex MAGPIX© (Luminex Corp, Austin, TX). For serological data analysis, infants under 1 year of age were excluded from each dataset to remove any influence of maternally derived antibodies [59]. Antibody responses measured as median fluorescence intensity (MFI) values were normalised against the MFI values of the positive control run on each plate. For each plate, the percentage of plate-to-reference standard MFI difference was calculated and used to adjust the median MFI values.

### Statistical analysis

All statistical analyses were conducted in Stata IC 15 (Stata Corp, College Station, TX, USA). A cut-off for seropositivity was determined based on finite mixture models according to the mean of log MFI values plus three standard deviation of the seronegative population. Separate cut-off values were generated for each antigen [60]. Individuals were categorised as seropositive for each species if their antibody responses were above the cut-off for either of the two or five antigens for *P. falciparum* and *P. vivax*, respectively. SCR were estimated by fitting a reverse catalytic model to seroprevalence data for each species [59]. Models allowing two forces of infection in SCR were fitted if deemed a better fit, using likelihood ratio methods. Mixed effects logistic regression models were performed to examine risk factors associated with being seropositive to *P. vivax*. Variables with evidence of an association (*p* < 0.05) in bivariate analysis were included in a multivariable model. Health facility was treated as a random effect variable in both bivariate and multivariable models.

### Spatial analysis

The ‘Normal model’ in the spatial software SaTScan (v.9.4.2) was used to detect clusters of individuals with higher than average age-adjusted antibody responses to each antigen per survey. In order to obtain age-adjusted values, the MFI data were log10 transformed and the residuals from linear regression were used to determine whether antibody responses were higher or lower than expected for any given age assuming a homogeneous distribution of risk across age. Firstly, residuals were categorised into four categories, i.e. below 25th percentile, 25th–75th percentile, 75th–90th percentile and above 90th percentile for each antigen. Individuals were then assigned score 4 (highest) if they had residual values above the 90th percentile, 3 (higher than average) for 75–90th percentile, 2 (average) for 25–75th percentile and 1 (low) for residual below the 25th percentile to any of the two or five antigens for *P. falciparum* or *P. vivax* antigen, respectively. The residual scores were then used to calculate non-overlapping, statistically significant (*p* < 0.05) clusters of higher than average age-adjusted antibody responses with a maximum radius of 3 km, minimum 2 observations detected in a cluster using the Purely Spatial scan. The analysis was run separately for each survey to ascertain spatial pattern at each survey time point. Clusters identified from SatScan were then plotted in QGIS software (v.3.6.3) to identify the potentially receptive areas. Spatial autocorrelation for each survey time point was assessed using Moran’s I in ArcGIS (v.10.5) using the age-adjusted antibody residuals from the regression model.

## Results

### Study enrolment and population demographics

A total of 9453 individuals were sampled during four repeated cross-sectional surveys performed in eight health facilities in Kulon Progo District, Yogyakarta Province, Indonesia, during the period of May 2017 to April 2018 (Table 1). Blood smears and dried bloodspot samples were collected from > 98% of attendees and their companions. Participation rates were above 90% for all surveys, ranging from 82 to 100% across facilities. Study participants were mostly female (65%), the median age was 42 years old (IQR 27–55), and the majority attended the facilities as patients (78.6%). Children were underrepresented in the sample, in comparison to the general population. Approximately 30% of the study population were forest workers involved in coconut/palm tapping, fruit farming, logging and other related jobs. A total of 42% of the study population reported having at least one bed net in their house, resulting in overall usage of 27% in the study population. Only 16% of the population reported recent travel, with the highest proportion of travel recorded during quarters 1 and 2 (May to October 2017). Approximately 5% of the study population were febrile or reported having fever in the previous 24 h.

**Table 1:** Number of samples, participation rates and general characteristics of health facility attendees per survey

	Quarter 1	Quarter 2	Quarter 3	Quarter 4	Total
	(May–July)	(August–October)	(November–January)	(February–April)	
Sample size, *n*	2363	2370	2379	2341	9453
*n* per facility					
Kokap 1	299	300	286	300	1185
Kokap 2	298	298	297	301	1194
Samigaluh 1	298	300	298	299	1195
Samigaluh 2	300	297	300	280	1177
Kalibawang	296	300	298	263	1157
Girimulyo 1	285	299	300	299	1183
Girimulyo 2	300	276	300	299	1175
Pengasih 2	287	300	300	300	1187
Participation rates					
Mean %	95	96	91	96	94
Range*	91–99	90–99	82–99	90–100	91–96
Female, *n* (%)	1578 (66.8)	1527 (64.4)	1530 (64.3)	1502 (64.2)	6137 (64.9)
Age, median (IQR)	40 (25–54)	41 (27–54)	42 (27–55)	43 (30–57)	42 (27–55)
Patients, *n* (%)	1803 (76.3)	1939 (81.8)	1878 (78.9)	1812 (77.4)	7432 (78.6)
Occupation, *n* (%)					
Forest workers	655 (27.7)	709 (29.9)	620 (26.1)	800 (34.2)	2784 (29.5)
Non-forest workers	685 (29.0)	647 (27.3)	738 (31.0)	678 (29.0)	2748 (29.1)
Not working	1023 (43.3)	1014 (42.3)	1021 (42.9)	859 (36.8)	3917 (41.5)
Lives in a house with bed net, *n* (%)	1091 (46.2)	1132 (47.8)	999 (42.0)	777 (33.3)	3999 (42.3)
Slept under the bed net, *n* (%)	710 (30.1)	685 (28.9)	666 (28.0)	527 (22.5)	2588 (27.4)
Recent travel, *n* (%)	595 (25.2)	581 (24.6)	211 (8.9)	111 (4.7)	1498 (15.9)
Fever, *n* (%)	127 (5.4)	116 (5.0)	146 (6.1)	93 (4.0)	484 (5.2)

*Range of health facility-level summaries

### Data captured by routine passive surveillance during the study period

The routine passive and reactive case detection in the study area detected 72 *P. vivax* and 8 *P. falciparum* microscopy-positive infections out of 15,067 slides read in 2017, with the majority of infections found in males (70.2%) and adults over 15 years old (89.0%). All *P. falciparum* infections were classified as imported. The majority of the *P. vivax* infections (86.1%, *n* = 62) were found in Kokap 1 health facility catchment area in quarter 2 (74%, *n* = 46). Of all of the infections detected, 39% (*n* = 24) were detected passively at the health facility, with the rest being detected via door to door active case detection performed by the village malaria workers (i.e. screening of suspected cases based on clinical signs). The *P. vivax* cases found through active case detection in Kokap 1 area were classified as a malaria outbreak by local authorities as there had been no indigenous case reported in the area since 2016, with only 2 *P. vivax* relapsed cases reported in July 2017.

### Health facility-based serological surveillance

**Fig. 2:**
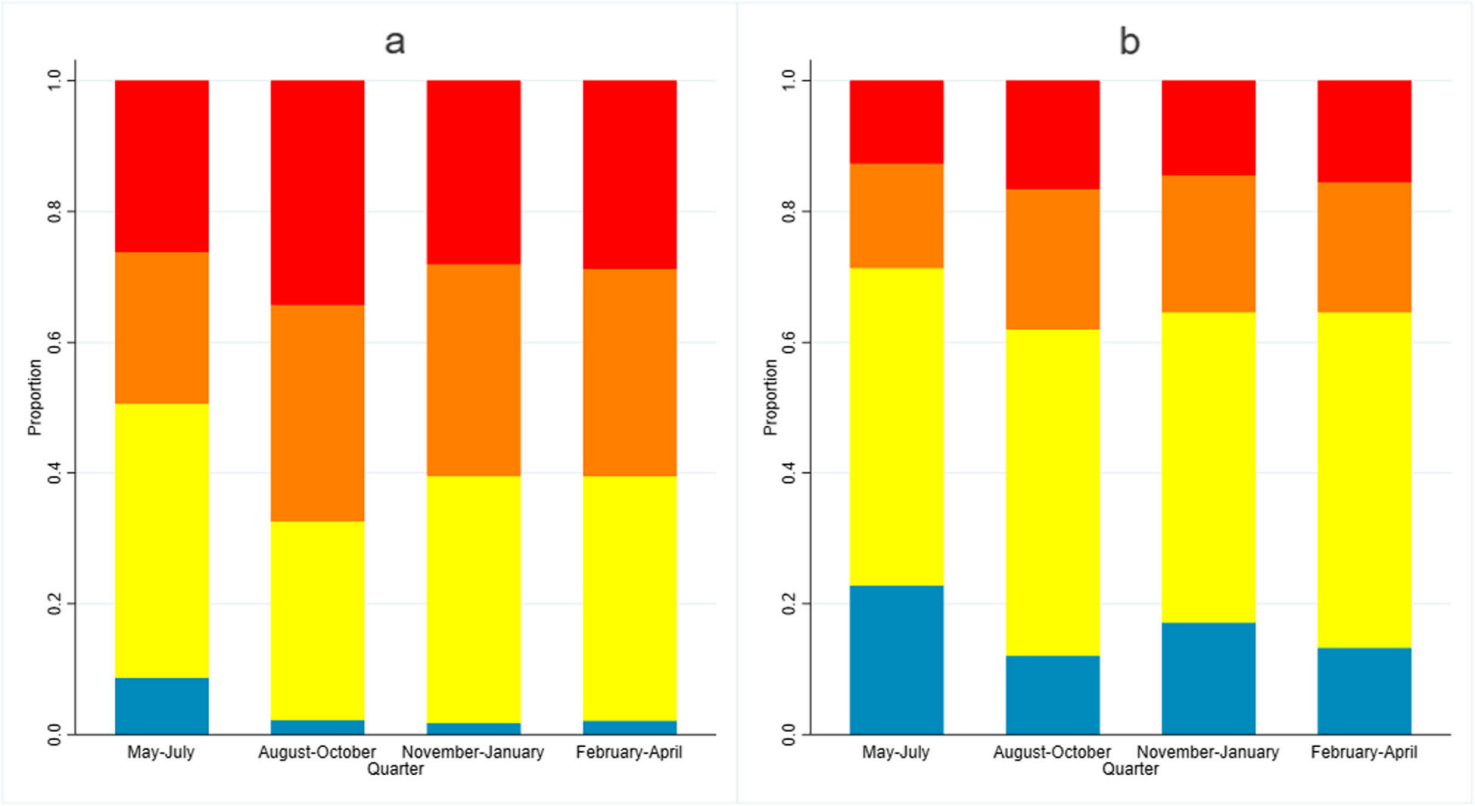
Proportion of individuals based on score of age-adjusted antibody responses to multiple **a** *P. vivax* and **b** *P. falciparum* antigens by survey time. Blue represents proportion of individuals with low age-adjusted antibody responses (score 1), yellow represents average (score 2), orange represents higher than average (score 3) and red represents proportion of individuals with highest age-adjusted antibody responses (score 4)

Few microscopy-positive infections were detected; 6/9356 (0.06%, 95% CI 0.03–0.14) for *P. vivax* and no *P. falciparum-*positive individuals were identified. All infections were found in Kokap 1 health facility, with 5 infections detected in quarter 2 and 1 in quarter 4. Of these infections, 1 was from a companion and 5 were from patients not suspected of having malaria. Most of the infections were asymptomatic (66.7%) (i.e. afebrile). Seroprevalence to *P. vivax* antigens was higher than seroprevalence to *P. falciparum* antigens in all surveys (Table 2). As expected, the seroprevalence increased with age for both species and varied between health facilities and over time. The highest overall seroprevalence was found in quarter 2 (August to October 2017), 46.3% (95% CI 44.2–48.3) and 23.9% (95% CI 22.2–25.7) for *P. vivax* and *P. falciparum*, respectively, with similar patterns observed according to a proportion of higher than average age-adjusted antibody responses to multiple antigens (Fig. 2).

**Table 2:** Seroprevalence to *P. vivax* and *P. falciparum* at quarterly surveys

	Quarter 1 (May–July)		Quarter 2 (August–October)		Quarter 3 (November–January)		Quarter 4 (February–April)	
	Number positive	Seroprevalence% (95% CI)	Number positive	Seroprevalence % (95% CI)	Number positive	Seroprevalence % (95% CI)	Number positive	Seroprevalence % (95% CI)
*P. vivax*								
Age group								
1–15 years old	44	12.1 (8.0–17.8)	24	11.2 (7.6–16.2)	26	17.6 (12.2–24.6)	9	10.5 (5.5–18.9)
> 15 years old	1014	41.0 (38.9–43.1)	1000	50.1 (47.9–52.3)	906	41.6 (39.6–43.7)	918	41.8 (39.8–43.9)
All ages	1058	38.8 (36.8–40.8)	1024	46.3 (44.2–48.3)	932	40.1 (38.2–42.1)	927	40.7 (38.7–42.7)
*P. falciparum*								
Age group								
1–15 years old	6	3.4 (1.6–7.5)	8	3.7 (1.9–7.3)	5	3.4 (1.4–7.9)	1	1.2 (0.2–7.8)
> 15 years old	405	18.8 (17.3–20.6)	521	26.1 (24.2–28.1)	489	22.6 (20.9–24.4)	504	23.0 (21.3–24.8)
All ages	411	17.7 (16.2–19.3)	529	23.9 (22.2–25.7)	494	21.4 (19.8–23.1)	505	22.1 (20.5–23.9)

### Transmission intensity and factor associated with transmission

**Fig. 3:**
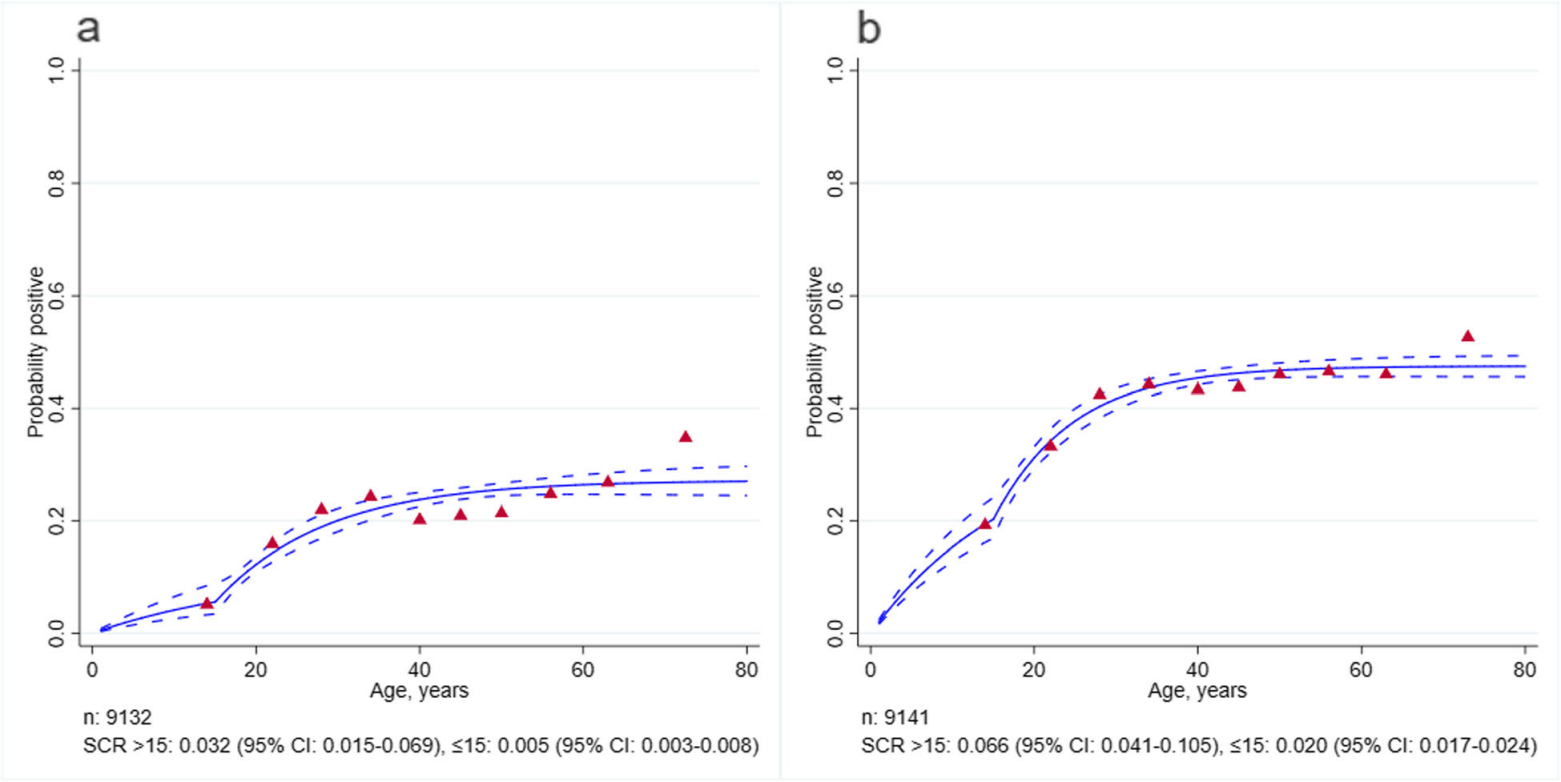
Age-seroprevalence plots for *P. falciparum* (**a**) and for *P. vivax* (**b**). Solid lines represent the fitted probability for being seropositive to either of the two or five antigens for *P. falciparum* and *P. vivax*, respectively*.* Dashed lines represent the 95% confidence interval of these fits and red triangles represent the observed proportion of seropositive per age decile. SCR value represents the average annual rate at which the population become seropositive to any of the *P. falciparum* or *P. vivax* antigen, respectively

**Fig. 4:**
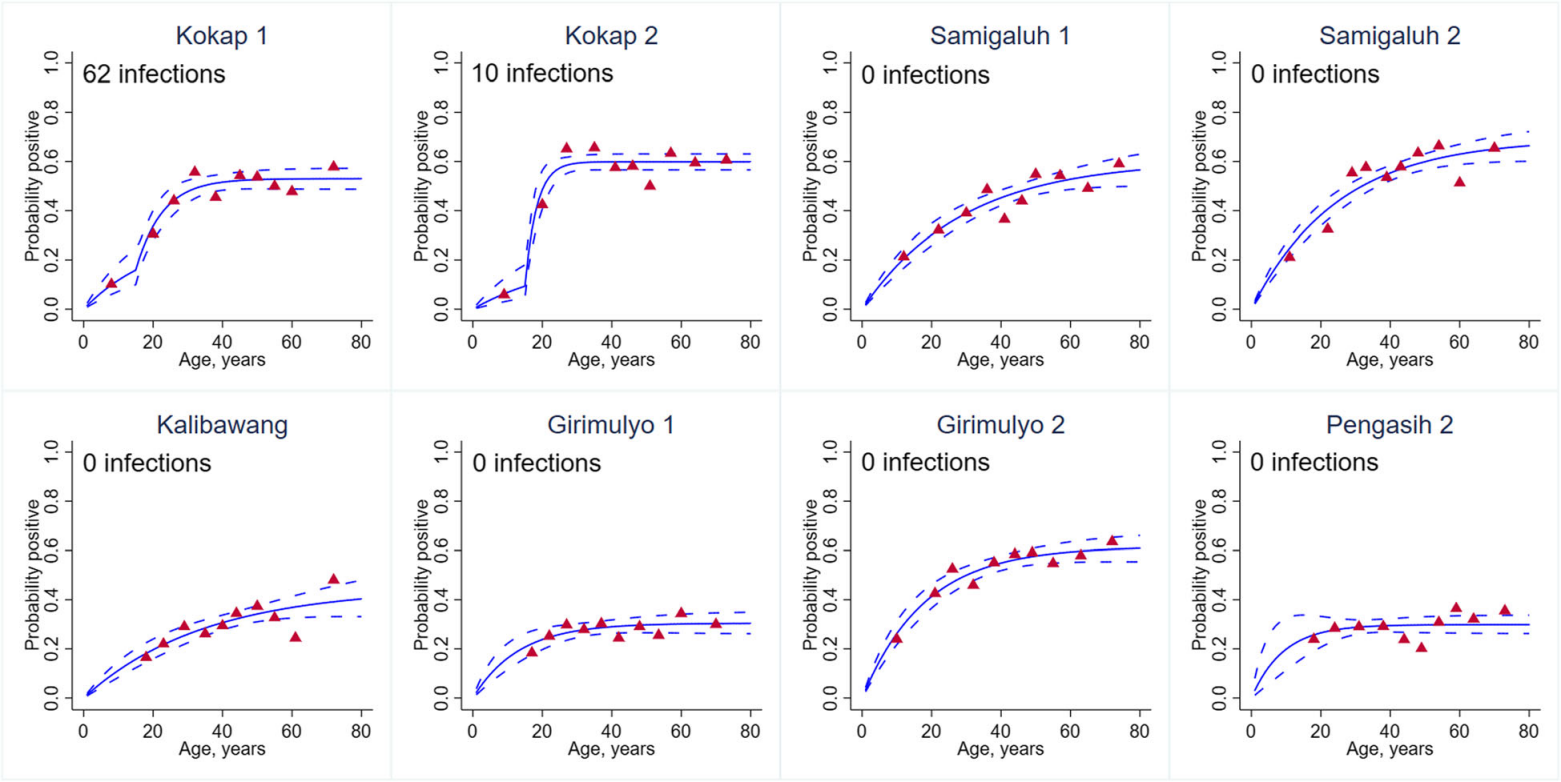
*P. vivax* age-seroprevalence plots and total number of *P. vivax* microscopy infections per health facility. Solid lines represent the fitted probability for being seropositive to either of the five *P. vivax* antigens*.* Dashed lines represent the 95% confidence interval of these fits and red triangles represent the observed proportion of seropositive per age decile. SCR value represents the average annual rate at which the population become seropositive to any of the *P. vivax* antigen

Based on the population-level SCR values, and consistent with microscopy and routine reporting data, the transmission intensity was higher for *P. vivax* than *P. falciparum.* The SCR model estimates (Fig. 3) suggested that there was evidence for two forces of infection. The *P. vivax* SCR was 0.020 person-year (95% CI 0.017–0.024) and 0.066 person-year (95% CI 0.041–0.105) for ≤ 15 and over 15 years old, respectively. The *P. falciparum* SCR was 0.005 person-year (95% CI 0.003–0.008) and 0.032 person-year (95% CI 0.015–0.069) for ≤ 15 and over 15 years old, respectively. At a health facility level, *P. vivax* SCR model estimates (Fig. 4) showed evidence for two forces of infection only in two health facilities where active cases were identified. However, a number of samples were low in the youngest age groups which may have influenced the fitting and estimates. Multivariable analysis found gender, occupation, time of survey and bed net use were significantly associated with being *P. vivax* seropositive, after controlling for other covariates factors (Table 3). The odds of being seropositive was higher in males (aOR 1.3, 95% CI 1.2–1.5), forest goers (aOR 1.2, 95% CI 1.0–1.3), those reporting sleeping under a bed net (aOR 1.2, 95% CI 1.1–1.3) and during quarter 2 (aOR 1.5, 95% CI 1.3–1.6).

**Table 3:** Factors associated with *P. vivax* transmission in Kulon Progo District, Indonesia, 2018

Variable	Total	*P. vivax* seropositive		Bivariate		Multivariable	
	*n* (%)	*n*	% (95% CI)	OR^a^ (95% CI)	*p*	aOR^b^ (95% CI)	*p*
Age (years)							
≤ 15	623 (6.8)	80	12.9 (10.5–15.7)	1		1	
16–30	2108 (23.1)	750	35.6 (33.6–37.6)	4.6 (3.6–5.9)	0.000	5.1 (3.9–6.6)	0.000
31–45	2531 (27.7)	1115	44.1 (42.1–46.0)	6.5 (5.0–8.3)	0.000	6.6 (5.1–8.7)	0.000
> 45	3880 (42.4)	1836	47.3 (45.8–48.9)	7.5 (5.9–9.7)	0.000	7.7 (5.9–10.0)	0.000
Gender							
Female	5945 (65.0)	2309	38.8 (37.6–40.1)	1		1	
Male	3206 (35.0)	1476	46.0 (44.3–47.8)	1.3 (1.2–1.4)	0.000	1.3 (1.2–1.5)	0.000
Status							
Accompanying	1960 (21.4)	895	45.7 (43.5–47.9)	1			
Patients	7191 (78.6)	2889	40.2 (39.0–41.3)	0.9 (0.8–1.0)	0.028		
Occupation							
Non-forest goers	2653 (29.0)	1023	38.6 (36.7–40.4)	1		1	
Forest goers	2685 (29.4)	1393	51.9 (50.0–53.8)	1.6 (1.4–1.8)	0.000	1.2 (1.0–1.3)	0.012
Unemployed	3810 (41.6)	1368	35.9 (34.4–37.4)	0.9 (0.8–1.0)	0.011	1.0 (0.9–1.1)	0.446
Survey time							
Quarter 1	2324 (25.4)	903	38.9 (36.9–40.9)	1		1	
Quarter 2	2217 (24.2)	1024	46.2 (44.1–48.3)	1.4 (1.2–1.5)	0.000	1.5 (1.3–1.6)	0.000
Quarter 3	2328 (25.4)	930	40.0 (38.0–42.0)	1.1 (0.9–1.2)	0.348	1.0 (0.9–1.2)	0.524
Quarter 4	2283 (24.9)	928	40.7 (38.6–42.7)	1.1 (1.0–1.2)	0.196	1.0 (0.9–1.2)	0.799
Bed net use							
No	6650 (72.7)	2556	38.4 (37.3–39.6)	1		1	
Yes	2502 (27.3)	1229	49.1 (47.2–51.1)	1.2 (1.1–1.3)	0.000	1.2 (1.1–1.3)	0.001
Fever							
No	8640 (94.9)	3604	41.7 (40.7–42.8)	1			
Yes	465 (5.1)	164	35.3 (31.1–39.7)	0.6 (0.5–0.8)	0.000		
Recent travel							
No	7681 (84.1)	3171	41.3 (39.3–44.4)	1			
Yes	1457 (15.9)	609	41.8 (40.2–42.4)	0.9 (0.8–1.1)	0.243		

^a^Bivariate OR adjusted by correlation at a health facility level

^b^Multivariable OR adjusted by other covariates with bivariate *p* value < 0.05, and correlation at a health facility level

Quarter 1: May–July 2017, Quarter 2: August–October 2017, Quarter 3: November 2017–January 2018, Quarter 4: February–April 2018

### Heterogeneity of transmission

**Fig. 5:**
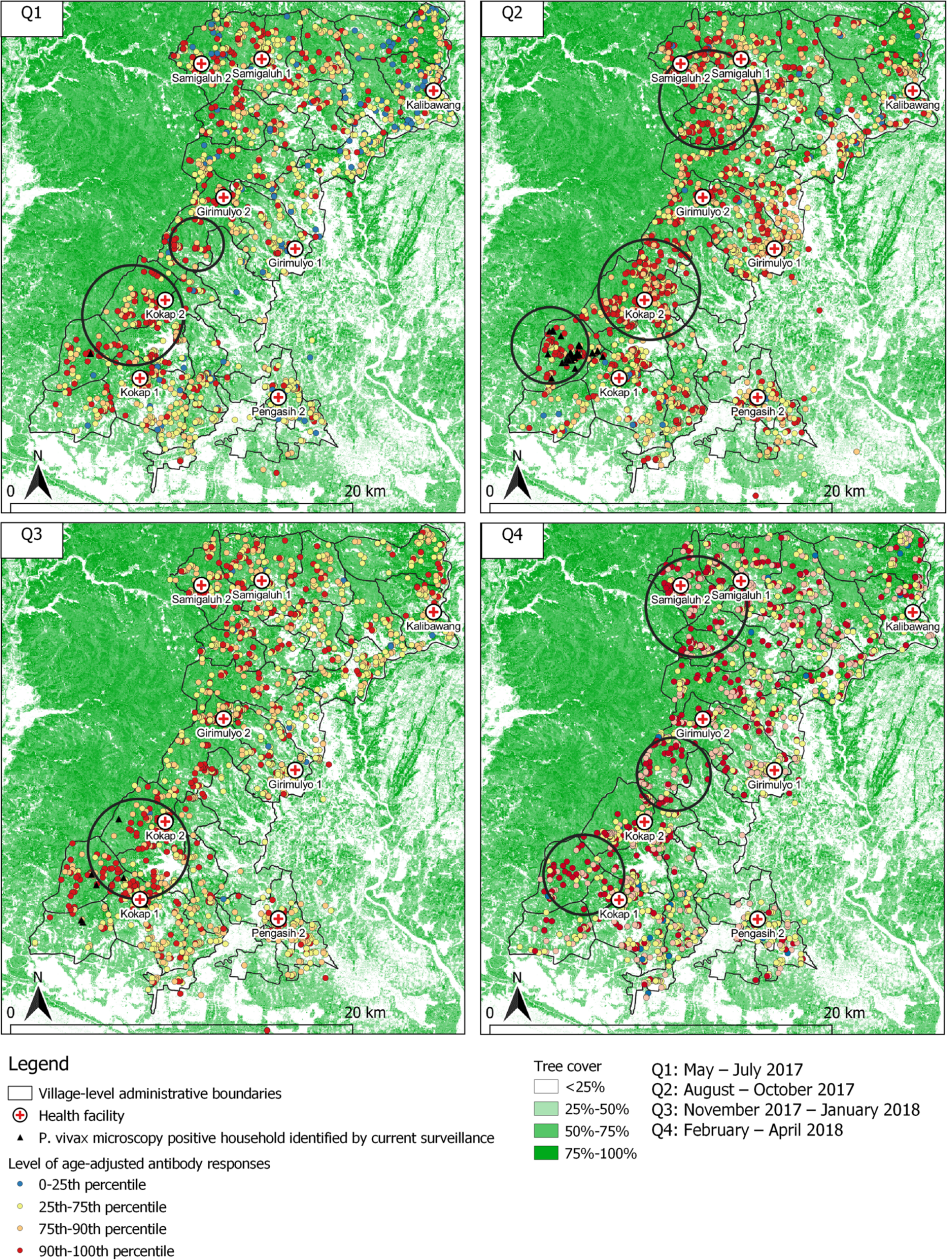
Spatial distribution of age-adjusted antibody responses to multiple *P. vivax* antigens over time of surveys overlaid with *P. vivax* microscopy infections captured by the current surveillance systems. Black triangles represent *P. vivax* microscopy-positive households. Black circle indicates a cluster of significantly higher than expected antibody responses detected using SaTScan (*p* value < 0.05)

Moran’s I suggested significant spatial autocorrelation for both species at each time point. The spatial analysis of higher than average age-adjusted antibody responses to multiple *P. vivax* antigens (Fig. 5) identified the same village in the Kokap 1 catchment area prior to when the *P. vivax* outbreak occurred during the quarter 2 (outbreak started in early August 2017, in between the first and second survey). The analysis consistently identified significant clusters of *P. vivax* exposure in catchment areas of Kokap 1 and Kokap 2 in each survey. These catchments were areas where active infections were detected by the existing surveillance in quarters 1, 2 and 3, with no cases in quarter 4. Significant clusters were also identified in Samigaluh 2 in quarters 2 and 4, and in Girimulyo 2 in quarter 4. The same areas were also identified using *P. falciparum* antigens (Additional file 1: Figure S1). In addition, the spatial analysis suggests that the *P. vivax* clusters identified were also the place where the majority of fever cases were seen in quarter 2 when the outbreak occurred (Additional file 2: Figure S2).

## Discussion

The study found that analysing serological and spatial epidemiological data collected via health facilities in quarterly cross-sectional surveys was a useful supplement to passive data collection and could potentially be used to identify and target areas that remain receptive to malaria, and therefore at risk of outbreaks (Additional file 3, 4, 5, and 6). Consistent with the parasitological data, the population-level SCR estimates suggest very low level of transmission in the ≤ 15-year-old population (current transmission). The SCRs equate to 5 per 1000 and 20 per 1000 people seroconverting per year for *P. falciparum* and *P. vivax*, respectively. For comparison, the SCRs in adults over 15 years old (historical transmission) were 32 per 1000 and 66 per 1000 people for *P. falciparum* and *P. vivax*, respectively. Moreover, spatial analysis of age-adjusted antibody responses identified clusters of high antibody responders in areas which subsequently report *P. vivax* cases. These findings support the potential utility of serological tools to improve malaria surveillance in the absence of active cases, and their incorporation in malaria elimination programmes. Multivariable analysis suggests that surveillance could potentially prioritise targeting males and forest goers as they were the high-risk populations who might reintroduce infections to a community in the future.

Although the accuracy of the mapping exercise varied within the eight health facilities (353–817 m), the addition of a relatively simple tablet-based participatory mapping approach with a short questionnaire administered during facility attendees’ interviews allowed the collection of fine-scale spatial variation of malaria infections and exposure. If employed, this approach could iteratively improve spatial accuracy of public health mapping at the local level [30]. Integrating spatial data with age-adjusted antibody responses to a panel of malaria antigens identified health facility catchment areas with significantly higher antibody responses than the population average. These clusters of high antibody responses were detected in the same areas across all four surveys for both species and were the location for a malaria outbreak during the study period. Importantly, the serological outcomes highlighted the area prior to the outbreak and, had this area been subject to targeting with interventions or more in-depth surveillance, the outbreak may have been prevented. Areas that were most recently receptive to transmission could be targeted with interventions as these are places that may be most susceptible to outbreaks and this strategy is likely to be more efficient than untargeted approaches to reduce transmission in low transmission settings [5]. Two other clusters in Girimulyo 2 and Samigaluh 2 were identified, suggesting that other high-risk areas are located in the most forested areas of the region which also bordered with another malaria higher endemic setting with ongoing transmission [61].

Whilst the microscopy data collected during the repeated surveys identified very few infections, and therefore could not be utilised to identify risk factors, the numbers of serological positives enabled the examination of risk factors for exposure to infection within the population. Our analysis found that people who were *P. vivax* seropositive were threefold more likely to be *P. falciparum* seropositive. As there was no cross-reactivity evident from the serological data, this suggests that the population have been exposed to infections with both species, although this exposure could have been historical. This implies that both species are transmitted in similar areas and that these places are, or were, particularly receptive to the transmission of malaria. Risk factor analysis for *P. vivax* seropositivity confirmed that people aged over 15 years old, males and forest-related activities were associated with higher exposure to malaria. These findings are consistent with findings from previous studies in the area suggesting that malaria infection is expected to be less common among children compared to adults most likely due to a different level of behavioural risk (night outdoor activities and forest-related jobs such as loggers, coconut/palm tapper, fruit farmer, etc.) which leads to higher exposure among males and adults [7, 10, 31, 35]. Interestingly, higher exposure was also associated with bed net use. The coverage and usage of bed nets was relatively low in this study setting and may be indicative of people living in higher-risk areas being more likely to use a net, potentially due to the presence of more mosquitoes. The data suggest that people ≤ 15 years old were more likely to be sleeping under a bed net compared to adults over 15 years old. This finding may also suggest that a bed net is no longer effective to prevent transmission in the studied population. Therefore, an alternative intervention such as targeted repellent distribution for adults or impregnated hammocks for forest workers could be useful to reduce transmission in the future.

*P. vivax* seroprevalence was highest during the period of August to October. This overlaps with the expected high transmission season (August to December) and was also the period when people in the study were most likely to report recent travel. However, our analysis suggested that the clusters of high exposure identified in this study were not necessarily the place where recent travel from was reported. A possible explanation of these findings is that the transmission occurred after Ramadhan where people were more likely to return to their region after several days or weeks of travelling to areas of higher endemicity to gather and celebrate Eid day with their family. Previous studies indicated migration and high rates of imported cases from higher transmission areas as factors that linked to malaria resurgence and outbreaks in low transmission settings [10, 13, 31, 62]. A study in Zanzibar estimated that residents travelling to other endemic settings contribute 1 to 15 times more imported cases than visitors, highlight the importance of strengthening surveillance to capture infection in travellers in countries nearing elimination [63]. However, the investigation conducted by the surveillance program did not identify if there was a link between migration during or after Ramadhan with the outbreak occurred in the period. These findings suggest that surveillance needs to be intensified in periods with high population movement such as during and/or after Ramadhan and during fruit (i.e. durian) harvesting time which often coincides with the wet season in the region, to enable early detection and responses to prevent transmission in the future, particularly in receptive areas identified in the study.

Our findings suggest that serological analysis can be used to estimate heterogeneity of *P. falciparum* and *P. vivax* transmission and predict high-risk areas from a single health facility-based cross-sectional survey. This sampling approach could be a more efficient surveillance strategy as the serological sampling is performed (in addition to parasitological diagnosis) in well-established health infrastructures therefore allowing rapid treatment and surveillance response if clinical cases are detected. On the other side, the repeated surveys might potentially be more useful in informing short-term changes in malaria exposure in other endemic settings where malaria transmission is still ongoing and more intense.

Although the health facility surveys provide sufficient samples to estimate burden of infection and transmission level in the population, there were several limitations to be considered when implementing the methods. Firstly, we found that the facility survey approaches captured only a small proportion of children under 15 years of age compared to the general population. Whilst we have observed risk is significantly higher in adults and the underrepresentation of children may not be an issue for malaria in this setting, it could limit the approach for general disease surveillance. Routine data collected by the district health office surveillance suggest that this could be due to the low proportion of children attending public health facilities in some areas where private health facilities may be easier to access. This phenomenon might not be the case in many other countries where often young children are the most common demographic to attend health facilities. Future studies in Indonesia could consider attendees to private health facilities as an easy access group to improve the facility-based sampling approach. In addition, surveys based in facilities are likely to miss asymptomatic infections, as well as those occurring in people who choose not to use public facilities. This is indicated by our finding suggesting that majority of cases (61%) were captured by the active case surveillance. Secondly, people living further from facilities may be less likely to attend health facilities resulting in the methods being less likely to detect clusters of high exposure further from facilities. However, it is conceivable that iterative refinements of the maps over time with clinical and demographic data would improve this. Inclusion of a mapping exercise in active surveillance performed by community health workers would be useful to capture heterogeneity in areas further from the facilities or those not seeking care. It may also help to identify if there are any spatial aspects to specific movement and behaviours. Recent travel was not significantly associated with increased seropositivity, but being male and working in the forest were and, whilst there was some evidence of spatial autocorrelation in the data, this was not accounted for in the regression modelling meaning estimates are likely to be over-precise. There are potential benefits to understanding the spatial context for risk behaviours which may be influenced by season for farming or harvesting and for traditional and religious holidays. The fourth limitation is in the analysis and interpretation of the serological data. Whilst outwardly, the multiplex assay for serological screening is attractive in increasing the number of antigenic targets to both reduce the likelihood of missing individuals non-responsive to specific antigens and simultaneously screen for multiple species, the best analytical approaches in combining data are still relatively undeveloped and validated. Using standard approaches based on seroprevalence, SCR and regression analysis has generated important observations but in future it will be important to combine these into more readily usable metrics and/or platforms such as serological lateral flow devices that offers more rapid test [64].

## Conclusion

The health facility-based serological surveillance implemented and evaluated in this study provides an alternative approach for quickly obtaining parasitological, serological, geolocation and risk factor data. A single survey is efficient in supplementing the existing surveillance in very low endemic areas approaching zero cases, although the repeated surveys might be more useful in informing short-term changes in exposure in other higher endemic settings. Combining these methods with novel multiplex serological techniques could improve malaria surveillance capacity and result in a better understanding of transmission dynamics, in the absence of infection detected by standard diagnostic tools such as microscopy. Future work could expand the use of multiplex bead-based assays to include a panel of other species of plasmodium antigens as well as to other available neglected tropical diseases (NTDs) antigens such as soil-transmitted helminths and filariasis to similarly improve surveillance of these infections. How this approach is incorporated as a practical tool into programmes will require significant technological and operational refinement [65] and financial assessment of the potential benefit. However, the argument for serological surveillance is particularly strong for *P. vivax* as there are no current diagnostics to detect latent hypnozoites and this is what the approach described in the manuscript has detected. Finally, reliability of implementing these methods would need to be evaluated in other areas aimed at eliminating malaria. Future works will need to assess the bottleneck of implementing these methods to allow further integration into existing surveillance systems.

## Additional files

Additional file 1. Spatial distribution of age-adjusted antibody responses to multiple *P. falciparum* antigens over time of surveys overlaid with *P. vivax* microscopy infections captured by the current surveillance systems. Black triangles represent *P. vivax* microscopy positive households. Black circle indicates a cluster of significantly higher than expected antibody responses detected using SaTScan (*p* value < 0.05).

Additional file 2. Maps showing cluster of significantly higher than expected antibody responses to multiple *P. vivax* antigens over time of surveys overlaid with fever status and *P. vivax* microscopy infections captured by the current surveillance systems.

Additional file 3. Maps showing cluster of significantly higher than expected antibody responses to PvAMA-1 antigen over time of surveys overlaid with *P. vivax* microscopy infections captured by the current surveillance systems.

Additional file 4. Maps showing cluster of significantly higher than expected antibody responses to PvMSP-1-_19_ antigen over time of surveys overlaid with *P. vivax* microscopy infections captured by the current surveillance systems.

Additional file 5. Maps showing cluster of significantly higher than expected antibody responses to PfAMA1 antigen over time of surveys overlaid with *P. vivax* microscopy infections captured by the current surveillance systems.

Additional file 6. Maps showing cluster of significantly higher than expected antibody responses to PfMSP-1-_19_ antigen over time of surveys overlaid with *P. vivax* microscopy infections captured by the current surveillance systems.

## Abbreviations

CI: Confidence interval
PfAMA1: *P. falciparum* apical membrane antigen 1
PfMSP-1: *P. falciparum* merozoite surface protein 1
PvAMA-1-_19_: *P. vivax* apical membrane antigen 1
PvEBP: *P. vivax* erythrocyte binding protein
PvMSP-1: *P. vivax* merozoite surface protein 1
PvRBP1a: *P. vivax* reticulocyte binding protein 1a [amino acids 160–1170]
PvRBP2b: *P. vivax* reticulocyte binding protein 2b [amino acids 161–1454]
SCR: Seroconversion rates
WHO: World Health Organization
