# Supplementary figures and images for: Using health facility-based serological surveillance to predict receptive areas at risk of malaria outbreaks in elimination areas
Source: BMC Med. 2020 Jan 28;18:9. doi: 10.1186/s12916-019-1482-7 (PMC6986103; doi:10.1186/s12916-019-1482-7)

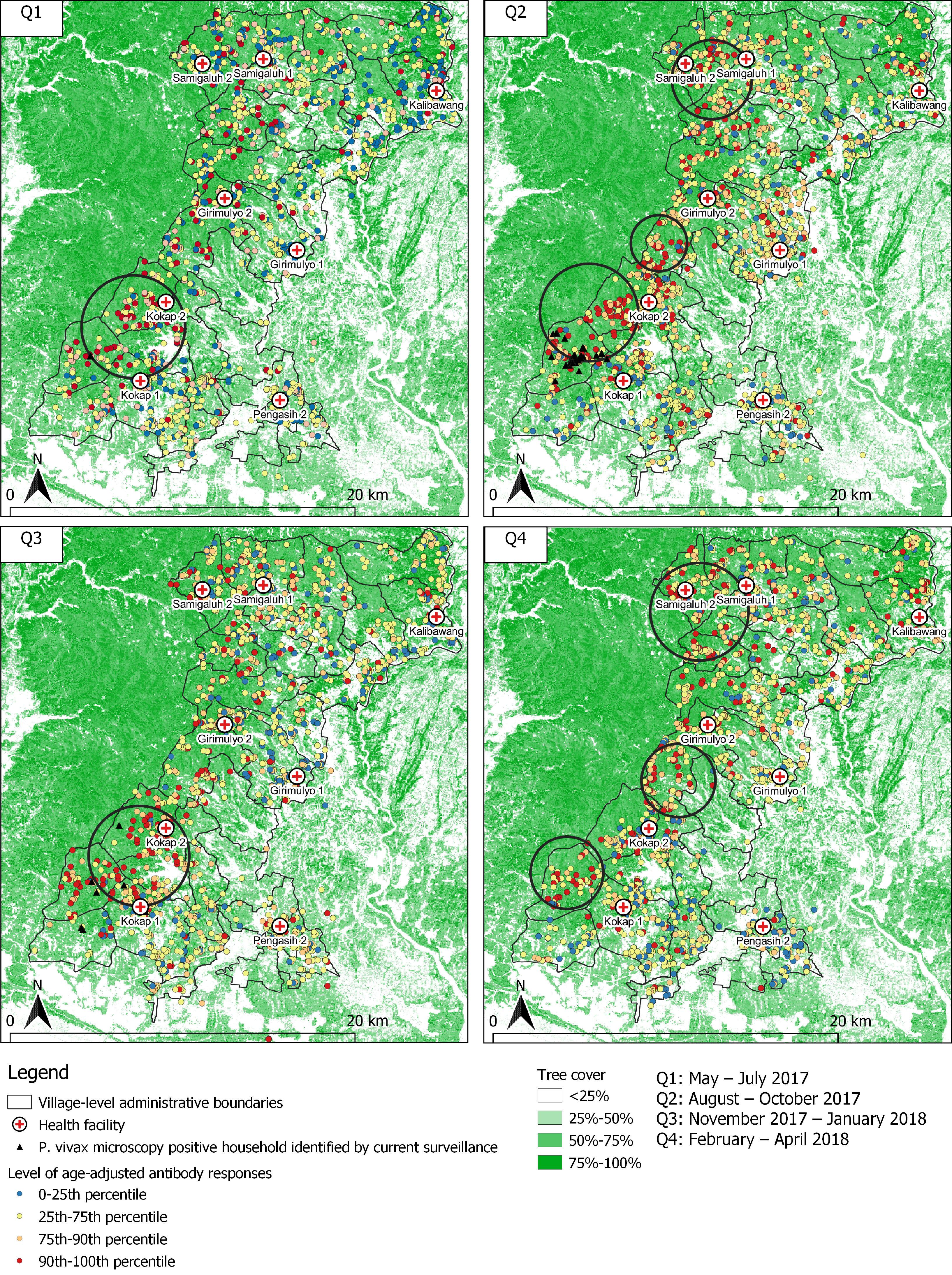

Supplement: Supplementary file 1 — Spatial distribution of age-adjusted antibody responses to multiple P. falciparum antigens over time of surveys overlaid with P. vivax microscopy infections captured by the current surveillance systems. Black triangles represent P. vivax microscopy positive households. Black circle indicates a cluster of significantly higher than expected antibody responses detected using SaTScan (p value < 0.05). [file 12916_2019_1482_MOESM1_ESM.png]

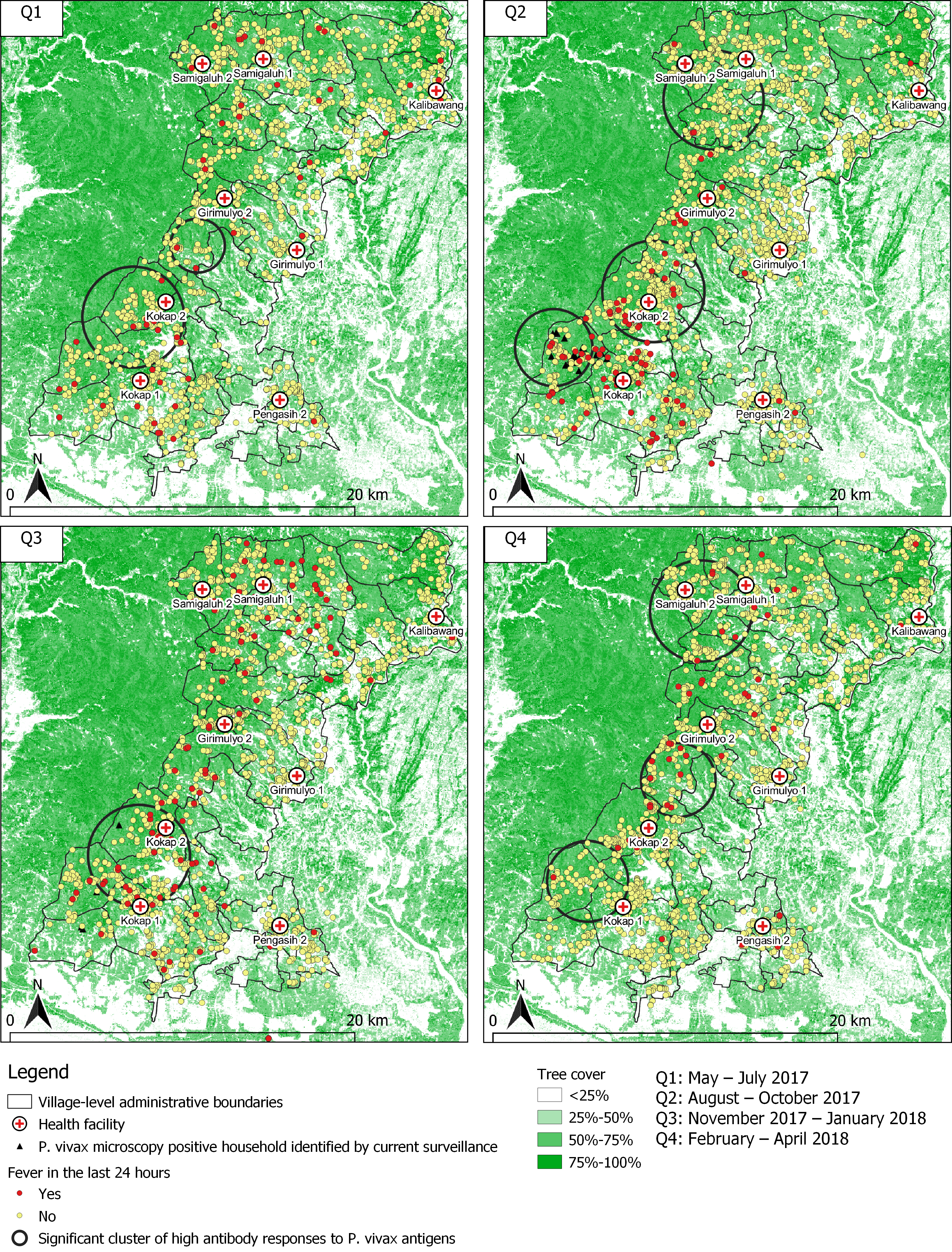

Supplement: Supplementary file 2 — Maps showing cluster of significantly higher than expected antibody responses to multiple P. vivax antigens over time of surveys overlaid with fever status and P. vivax microscopy infections captured by the current surveillance systems. [file 12916_2019_1482_MOESM2_ESM.png]

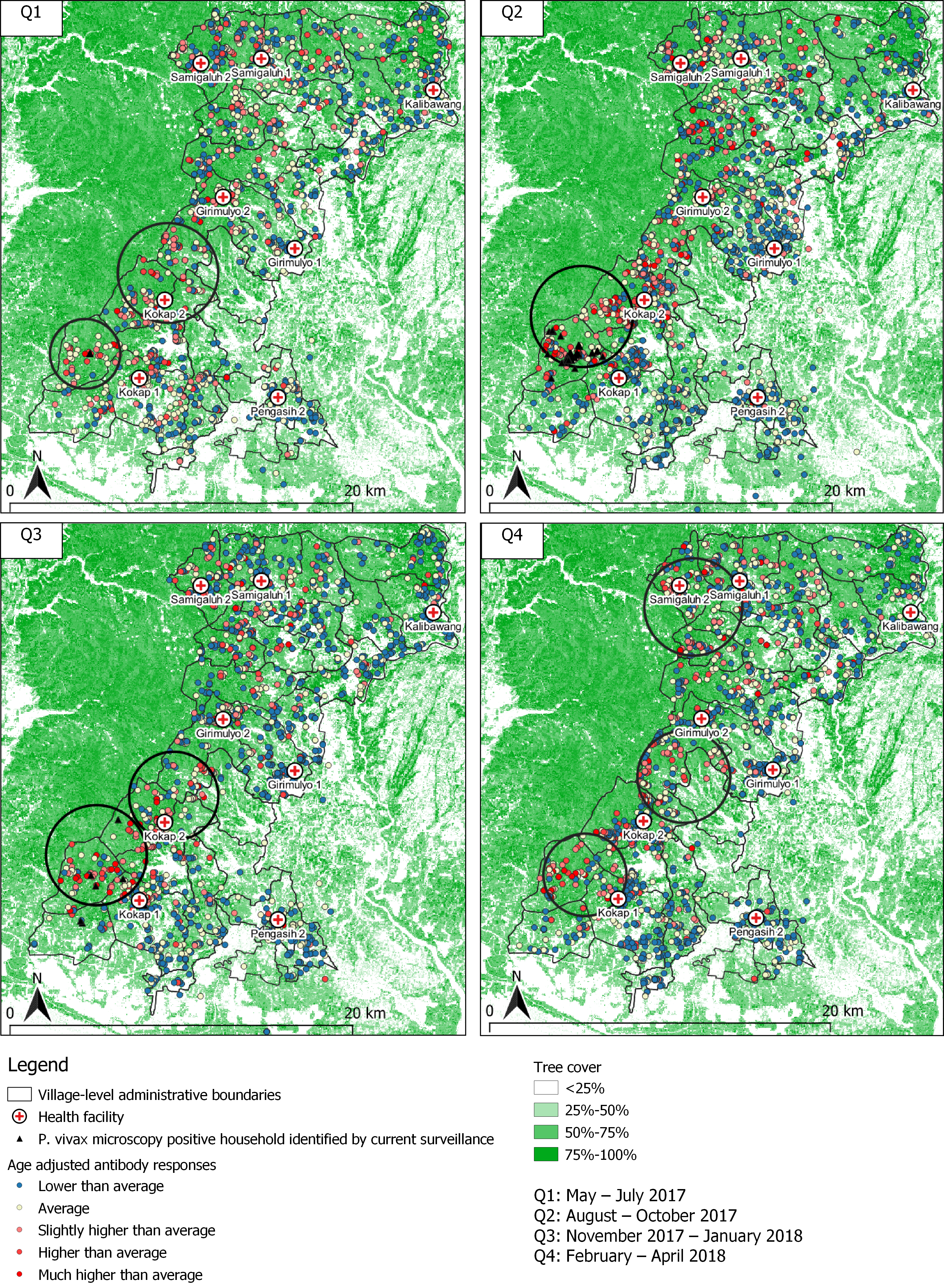

Supplement: Supplementary file 3 — Maps showing cluster of significantly higher than expected antibody responses to PvAMA-1 antigen over time of surveys overlaid with P. vivax microscopy infections captured by the current surveillance systems. [file 12916_2019_1482_MOESM3_ESM.png]

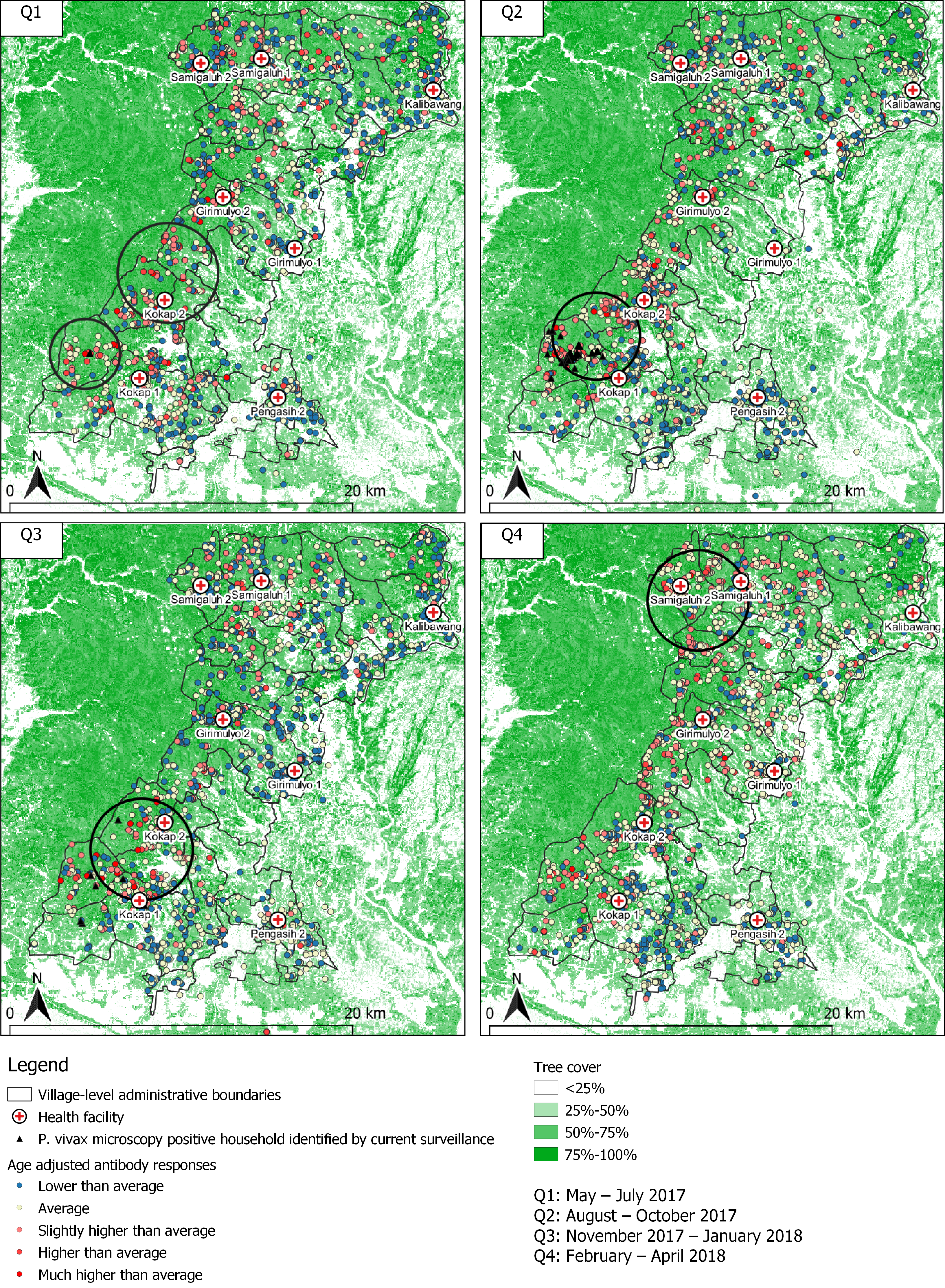

Supplement: Supplementary file 4 — Maps showing cluster of significantly higher than expected antibody responses to PvMSP-1-19 antigen over time of surveys overlaid with P. vivax microscopy infections captured by the current surveillance systems. [file 12916_2019_1482_MOESM4_ESM.png]

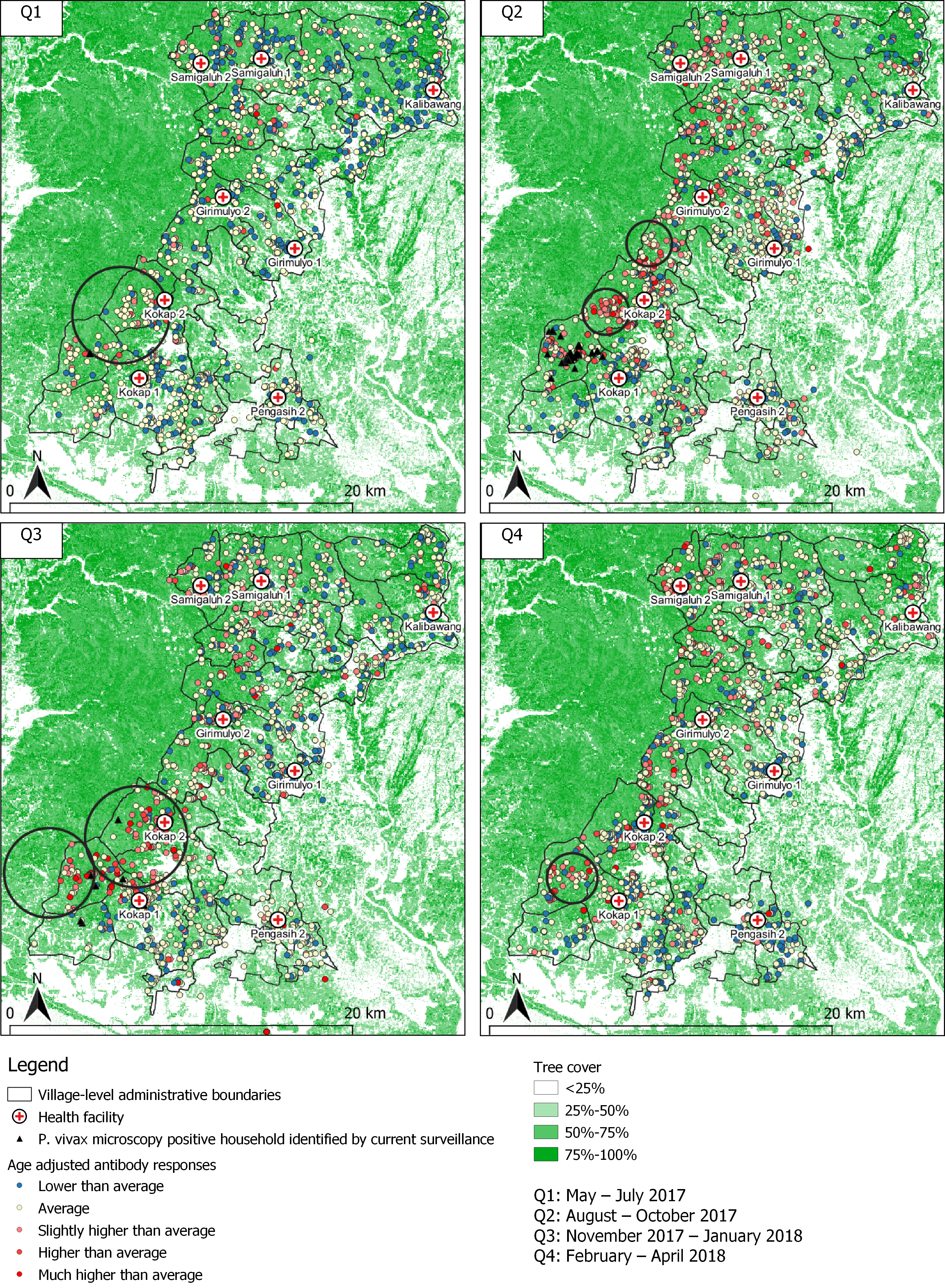

Supplement: Supplementary file 5 — Maps showing cluster of significantly higher than expected antibody responses to PfAMA1 antigen over time of surveys overlaid with P. vivax microscopy infections captured by the current surveillance systems. [file 12916_2019_1482_MOESM5_ESM.png]

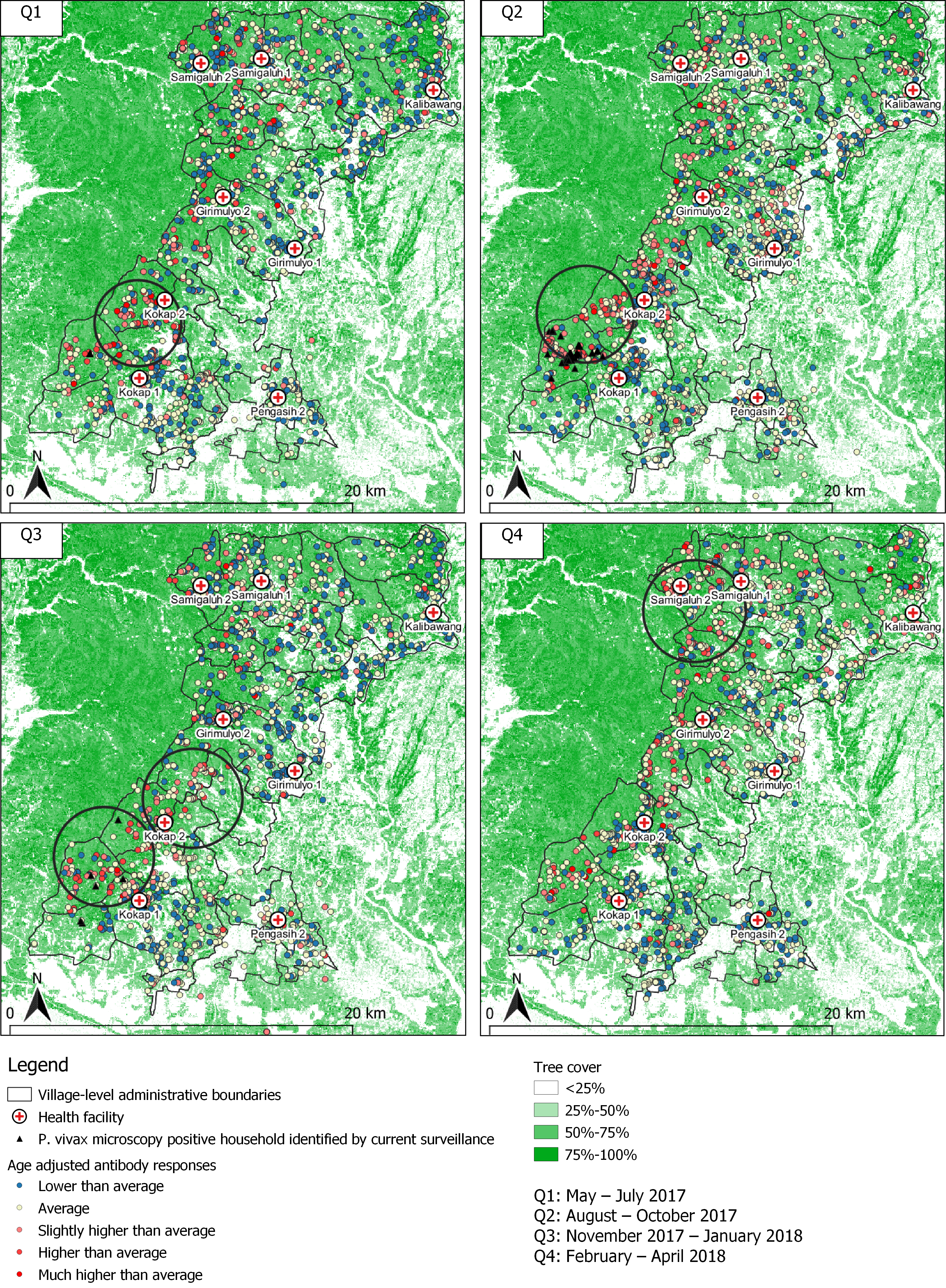

Supplement: Supplementary file 6 — Maps showing cluster of significantly higher than expected antibody responses to PfMSP-1-19 antigen over time of surveys overlaid with P. vivax microscopy infections captured by the current surveillance systems. [file 12916_2019_1482_MOESM6_ESM.png]
